# Resolution of *ab initio* shapes determined from small-angle scattering

**DOI:** 10.1107/S2052252516016018

**Published:** 2016-10-27

**Authors:** Anne T. Tuukkanen, Gerard J. Kleywegt, Dmitri I. Svergun

**Affiliations:** a EMBL Hamburg c/o DESY, European Molecular Biology Laboratory, Notkestrasse 85, 22607 Hamburg, Germany; b European Bioinformatics Institute (EMBL–EBI), European Molecular Biology Laboratory, Welcome Genome Campus, Hinxton, Cambridge CB10 1SD, England

**Keywords:** small-angle scattering, *ab initio* modelling, spatial resolution

## Abstract

Spatial resolution is an important characteristic of structural models, and the authors of structures determined by X-ray crystallography or electron cryo-microscopy always provide the resolution upon publication and deposition. Small-angle scattering of X-rays or neutrons (SAS) has recently become a mainstream structural method providing the overall three-dimensional structures of proteins, nucleic acids and complexes in solution. However, no quantitative resolution measure is available for SAS-derived models, which significantly hampers their validation and further use. Here, a method is derived for resolution assessment for *ab initio* shape reconstruction from scattering data. The inherent variability of the *ab initio* shapes is utilized and it is demonstrated how their average Fourier shell correlation function is related to the model resolution. The method is validated against simulated data for proteins with known high-resolution structures and its efficiency is demonstrated in applications to experimental data. It is proposed that henceforth the resolution be reported in publications and depositions of *ab initio* SAS models.

## Abstract 2

A quantitative resolution measure of the *ab initio* shapes restored from small-angle scattering data is introduced based on the variability of multiple reconstructions. The new measure is validated in simulated examples and its efficiency has been demonstrated in applications to experimental data.

## 1. Introduction   

**Figure 1:**
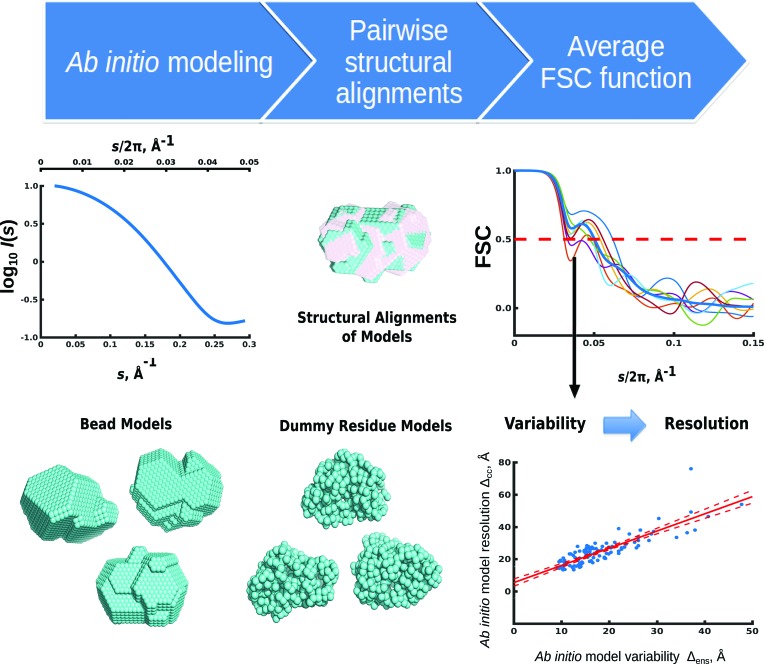
Overview of the FSC approach for estimating the variability of structural ensembles. Firstly, multiple runs of *ab initio* modelling, shown here for lysozyme, are performed to generate an ensemble of models from the given scattering intensity profile *I*(*s*) (*s* = 4πsinθ/λ, where 2θ is the scattering angle and λ is the radiation wavelength). The reconstructed bead or dummy-residue models are then structurally aligned and their pairwise FSC functions are computed. The average of all pairwise FSC functions is used to determine the variability estimate Δ_ens_ as 2π/*s*_ens_, where *s*_ens_ is the momentum-transfer value at which the average FSC falls below 0.5. The corresponding resolution is estimated based on the variability using a linear regression model.

In small-angle scattering (SAS) studies, the nanostructure of matter is probed using X-rays or neutrons. The technique, which is applicable to completely or partially disordered objects, is particularly useful for the study of biological macromolecules in close to native solutions and medical formulations (Svergun *et al.*, 2013 ▸). In a SAS experiment, scattering intensity *I* from a dilute solution of macromolecules (*e.g.* proteins, nucleic acids or complexes) is recorded. Since the molecules are randomly oriented, the scattering profiles from individual particles are averaged, yielding an isotropic intensity *I*(*s*) as a function of momentum transfer *s* = 4πsinθ/λ, where 2θ is the scattering angle and λ is the radiation wavelength (Fig. 1 ▸). Nonetheless, even from these one-dimensional scattering profiles one can reconstruct three-dimensional models of the overall particle structure. This can be performed either without any additional information by *ab initio* approaches (Chacón *et al.*, 1998 ▸; Svergun, 1999 ▸, 2001 ▸; Franke & Svergun, 2009 ▸), or by hybrid modelling utilizing known atomic structures of domains or subunits to construct models of complexes (Petoukhov & Svergun, 2005 ▸).

The possibility of obtaining three-dimensional models from solution-scattering profiles, accompanied by the recent advances in instrumentation that are paving the way for high-throughput studies on modern synchrotrons (Pernot *et al.*, 2013 ▸; Acerbo *et al.*, 2015 ▸; Blanchet *et al.*, 2015 ▸; Bizien *et al.*, 2016 ▸), have boosted the popularity of SAS in structural biology during the last decade. The resurgence of SAS in structural biology is shown by an approximately fourfold increase in publications devoted to biological solution scattering in the last ten years according to PubMed statistics. Still, two major unresolved issues prevent the method from becoming a fully established structural biology technique. On one hand, given that the amount of information in SAS data is limited by spherical averaging, reconstructing three-dimensional structural models is inherently ambiguous and ensembles of models may be obtained that fit the experimental data equally well. On the other hand, being a low-resolution method, SAS does not provide information at an atomic level. Usually, SAS-based models are tacitly assumed to have a resolution of ∼10–20 Å, but no objective measures of resolution are available for them. This situation contrasts with structural techniques such as macromolecular X-ray crystallography (MX) and electron cryo-microscopy (EM), where the resolution criteria are well established.

For MX, the resolution depends according to Bragg’s law on the reciprocal of the highest order diffraction peak that can be detected from the background and that enters structure refinement (Bragg & Bragg, 1913 ▸). Application of this principle to deduce the resolution from the maximum momentum-transfer value *s*_max_ that was used to generate a SAS model would only give a nominal theoretical limit (2π/*s*_max_) without much practical value because of the ambiguity of SAS reconstructions. The resolution of EM data is commonly estimated by a Fourier shell correlation (FSC) method, where the electron-density maps reconstructed from two separately processed sets of experimental images are compared in reciprocal space (van Heel & Stöffler-Meilicke, 1985 ▸; Saxton & Baumeister, 1982 ▸; Harauz & van Heel, 1986 ▸). A separation into two independent data sets is obviously not applicable to SAS, where only a single experimental scattering profile is available, and the experimental SAS data are inter-correlated owing to Shannon sampling (Shannon & Weaver, 1949 ▸). Another major structural method, nuclear magnetic resonance spectroscopy (NMR), yields ensembles of atomic models compatible with the experimental NMR data. Although no agreed resolution criteria for NMR models are available [*e.g.* the root-mean-square-deviation (r.m.s.d.) between the models may sometimes overestimate the resolution; Montelione *et al.*, 2013 ▸; Vuister *et al.*, 2014 ▸], stereochemical validation can help one to assess the model quality. Such a validation-based approach, also common for MX structures (Read *et al.*, 2011 ▸; Doreleijers *et al.*, 2012 ▸; Berjanskii *et al.*, 2012 ▸), cannot be used for solution scattering as SAS models do not reveal atomic detail.

In *ab initio* shape reconstruction using SAS data, the three-dimensional models are represented using finite volume elements, *e.g.* densely packed beads (Svergun, 1999 ▸; Franke & Svergun, 2009 ▸) or dummy residues (DRs; Svergun *et al.*, 2001 ▸). The reconstruction starts from a random configuration of volume elements and utilizes an optimization algorithm (*e.g.* Monte Carlo-based simulated annealing) to fit the computed theoretical scattering from the model to the experimental SAS profile. In addition to the discrepancy of the fit, the target function includes constraints ensuring the physical feasibility of models such as interconnectivity and compactness. Multiple *ab initio* reconstructions starting from different random configurations yield varying models with similar overall appearance, with each model being consistent with the experimental SAS data. Typically, ten to 20 reconstructions are performed, and the models (including their enantiomorphs, which give exactly the same scattering patterns) are superimposed using pairwise normalized spatial discrepancies (NSDs; Kozin & Svergun, 2001 ▸). The model (or enanthiomorph) with the smallest average NSD is selected, the other models are aligned with it and the resulting map is averaged. The average model has been demonstrated to retain the most persistent features of all reconstructions (Volkov & Svergun, 2003 ▸). However, the averaging procedure provides no assessment of resolution as, in contrast to NMR ensembles, multiple SAS models do not possess a one-to-one atom or residue correspondence and the calculation of r.m.s.d. values is not possible.

The lack of objective criteria to assess SAS model resolution is a serious drawback that hinders critical assessment of the results, especially in view of the growing use of SAS in structural biology and the deposition of SAS models in archives (Hura *et al.*, 2009 ▸; Valentini *et al.*, 2015 ▸). A quantitative resolution measure is needed for meaningful comparison of SAS models with structural results obtained by other experimental techniques. Here, we present an approach to estimate the resolution of *ab initio* SAS-derived shapes by analysing FSC functions across an ensemble of models compatible with the SAS data. It is demonstrated that the average FSC function over an ensemble that reflects the variability of models can be related to the resolution of the individual models in the shape reconstruction. The approach is implemented in a publicly available computer program and its utility is demonstrated by a series of tests on synthetic data and by practical examples.

## 2. FSC measure of variability for SAS models   

Firstly, we introduce a variability measure of *ab initio* SAS models consisting of beads or DRs. Given that the numbering of volume elements in these models is arbitrary, no direct correspondence exists between the beads or DRs in two different models. A general real-space measure is thus difficult to define and the use of Fourier transforms is appropriate. Similar to the FSC function for EM (van Heel & Stöffler-Meilicke, 1985 ▸; Saxton & Baumeister, 1982 ▸; Harauz & van Heel, 1986 ▸), we employ the normalized cross-correlation coefficient between scattering amplitudes of two structural models over corresponding shells in reciprocal space. If *A* and *B* are two (appropriately aligned) bodies with known structures and *A*(**s**) and *B*(**s**) are their three-dimensional scattering amplitudes (here, **s** is the scattering vector in reciprocal space), a one-dimensional FSC is the function of the momentum transfer (also called spatial frequency),
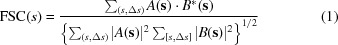
where (*s*, Δ*s*) are the radius and width of the spherical shell in reciprocal space. The scattering amplitudes of *ab initio* models *A* and *B* can be represented in reciprocal space using a spherical harmonics expansion, 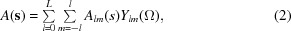


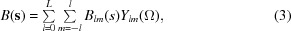
where **s** = (*s*, Ω) is the scattering vector in spherical coordinates, *A_lm_*(*s*) and *B_lm_*(*s*) are the partial scattering amplitudes of the models *A* and *B*, *L* is the truncation value which defines the accuracy of the expansion and *Y_lm_*(Ω) are the spherical harmonics of order (*l*, *m*) (Stuhrmann, 1970 ▸). The partial amplitudes are computed using the form factor *f_k_*(*s*) of either a bead or a dummy residue and using the equation
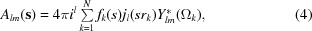
where *j_l_*(*sr*) are spherical Bessel functions. Substituting the spherical harmonics representation of scattering amplitudes [equations (2) and (3)] into (1) and using the orthogonality of spherical harmonics functions 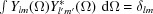, the FSC function becomes
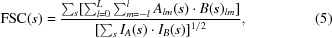
where *I_A_*(*s*) and *I_B_*(*s*) are the scattering intensities of the models *A* and *B*.

For non-identical structures, the FSC decreases with momentum transfer, reflecting the loss of structural similarity with increasing resolution. FSC is commonly used in EM when comparing two density maps to estimate map resolution, which is defined as the spatial frequency at which the FSC function value falls below a certain threshold. The FSC function usually decreases monotonically, although local oscillations may also be observed, which can be dampened by selecting an appropriate width of the spherical shell Δ*s* [equation (1)].

As described above, multiple *ab initio* shape determinations are typically carried out in SAS, and an ensemble of (typically 10–20) aligned models, each consistent with the experimental data, is then available. To quantify the variability of an *ab initio* ensemble, the scattering amplitudes of the aligned models are computed and the pairwise FSC functions are evaluated using (1). The average of these FSC functions can then be used to obtain the variability measure Δ_ens_ of the ensemble (Fig. 1 ▸). For EM maps, the midpoint (0.5) threshold in the FSC function is often employed to define the resolution, although other values have also been discussed (van Heel & Schatz, 2005 ▸; Penczek, 2010 ▸). Our calculations on randomized models (§S1, Supporting Information) confirmed that the midpoint of the FSC does provide a resolution measure that agrees well with the randomization magnitude. An FSC threshold of 0.5 was therefore adopted in subsequent calculations and the variability measure Δ_ens_ was defined as 2π/*s*_ens_ from the momentum-transfer value *s*_ens_ at which the average FSC value falls below 0.5. Note that the averaging of the pairwise FSCs dampens the oscillations observed in the individual FSC functions, thereby improving the precision of *s*_ens_ estimation for the ensemble (Fig. 1 ▸).

### 2.1. Variability and the benchmark protein set   

**Figure 2:**
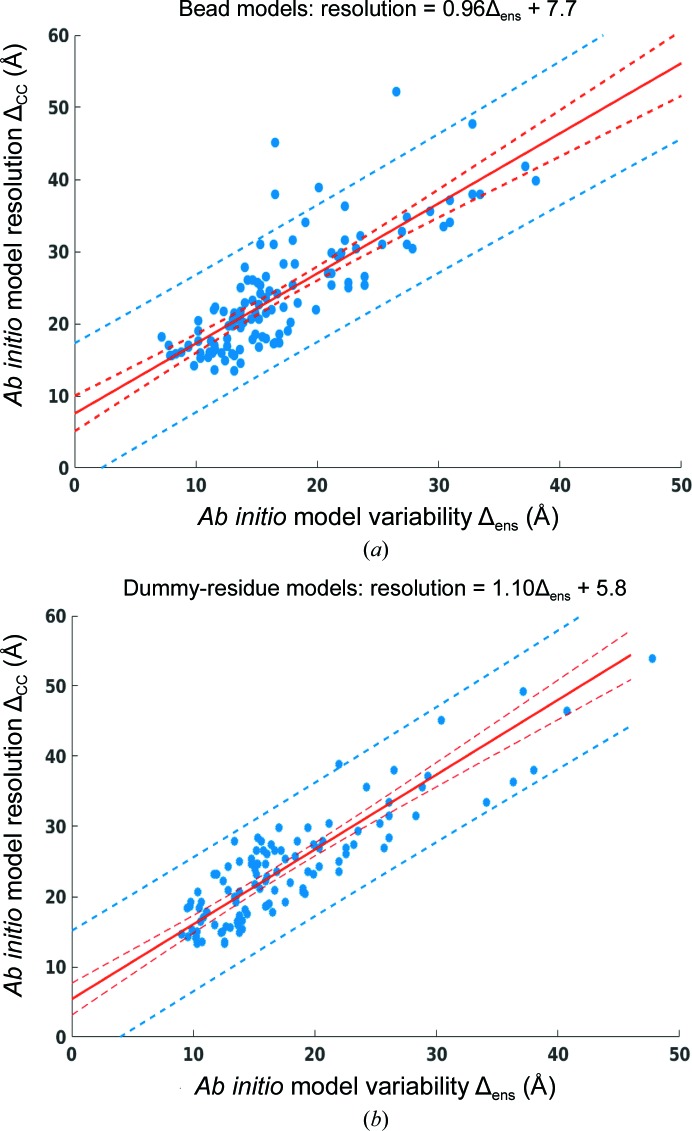
Relationship between the Δ_ens_ and Δ_CC_ values of the benchmark protein dummy-bead (*a*) and dummy-residue (*b*) ensembles. The two quantities show linear correlation for both bead (Pearson correlation coefficient *r* = 0.80) and dummy-residue (Pearson correlation coefficient *r* = 0.86) models. SAS resolution values can be estimated by linear regression models (bead models, resolution = 0.96Δ_ens_ + 7.7; dummy-residue models, resolution = 1.10Δ_ens_ + 5.8; red solid lines). The 95% confidence intervals are shown by red dotted lines and the 95% prediction intervals by blue dotted lines

To analyse the properties of the variability estimate Δ_ens_, we performed *ab initio* modelling on synthetic SAXS data sets from 107 benchmark proteins with a wide range of molecular weights (between 7 and 670 kDa), oligomeric states and SCOPe folds with known high-resolution structures from the Protein Data Bank (PDB; Fox *et al.*, 2014 ▸; Berman *et al.*, 2003 ▸; Supplementary Tables S1 and S2). The structures were annotated either as oblate, prolate or equant using Zingg’s shape classification of particles (Zingg, 1935 ▸). Synthetic SAXS profiles *I*(*s*) were generated for each benchmark with *CRYSOL* using spherical harmonics with order up to 18, a Fibonacci grid with order 17 and default parameters for the solvent density as well as for the contrast of hydration layers (Svergun *et al.*, 1995 ▸). This was followed by determination of the distance-distribution functions *p*(*r*) for each data set using *GNOM* (Semenyuk & Svergun, 1991 ▸) and shape reconstructions with *DAMMIF* (bead models) or *GASBOR* (DR models). Given that the range of scattering data containing overall shape information is inversely proportional to the particle size, the *DAMMIF* reconstructions were conducted in the range (0, 7.0/*R*_g_), where *R*_g_ is the radius of gyration of the protein. This low-angle portion of the scattering profile, corresponding to about five Shannon channels (Shannon & Weaver, 1949 ▸), contains overall shape information and this range is normally employed for shape determination. *GASBOR* reconstructions with more detailed dummy residue representation can utilize higher resolution data and were conducted using a fixed *s*_max_ value of 0.5 Å^−1^, which is a typical experimental SAS data range. For each protein, 20 reconstructions were generated, the models were pairwise aligned using *SUPCOMB* (Kozin & Svergun, 2001 ▸) and the variability within the ensembles was computed using the above FSC approach. Among the proteins in the benchmark set, the computed variability varied between 7.2 and 38.0 Å for the bead models and between 9.0 and 47.8 Å for the DR models (Fig. 2 ▸).

**Figure 3:**
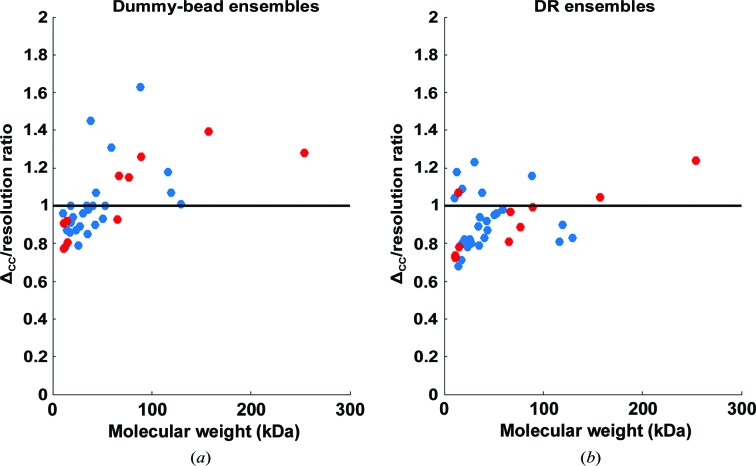
The ratio between the cross-validated resolution Δ_CC_ and the estimated SAS resolution for the jackknife set (blue dots) and for the experimental data set (red dots) as a function of the molecular weight for *DAMMIF* bead models (*a*) and *GASBOR* dummy-residue models (*b*).

Additionally, variable data ranges were used for a subset of 15 benchmark proteins such that the product *s*_max_·*R*_g_ equalled either 5.0, 7.0 or 9.0 (Supplementary Table S5). For *GASBOR* modelling, two fixed *s*_max_ values, 0.5 and 1.0 Å^−1^, were used. Furthermore, a set of experimental SAXS profiles was retrieved from the the Small Angle Scattering Biological Data Bank (SASBDB; Valentini *et al.*, 2015 ▸; Supplementary Table S9 and Fig. 3) to test the variability approach on typical experimental data ranges. 20 independent *ab initio* *DAMMIF* and *GASBOR* models were generated for every protein using the selected data ranges and default parameters of the programs.

## 3. From variability to resolution   

The ensemble variability is a measure of the reproducibility of the shape reconstruction, and on its own does not provide the resolution of the reconstructed models (which is a measure of their accuracy, *i.e.* how close they are to the ‘true structure’). A question arises as to whether and how the two quantities are related to each other. To answer this, we calculated the FSC functions between the known high-resolution X-ray crystallographic structures of the benchmark proteins and the *ab initio* models in the generated ensembles (Supplementary Tables S1 and S2 and Supplementary Figs. S1 and S2). The cross-correlated resolution Δ_CC_ of the ensembles is the actual resolution of the SAS models based on comparison with a known high-resolution structure of the same protein determined from the averaged pairwise FSC function at a cutoff of 0.5. For each *ab initio* model, the enantiomorph providing the better alignment with the reference atomic model from PDB was selected.

For all proteins analysed, Δ_CC_ was found to be systematically somewhat larger than the ensemble variability Δ_ens_ (Supplementary Tables S1 and S2 and Fig. 2 ▸). The Δ_CC_ values of the ensembles within the benchmark data set ranged between 13.5 and 52.2 Å for bead models and between 13.4 and 76.0 Å for DR models. Most importantly, the variability measure Δ_ens_ and the cross-validated resolution Δ_CC_ demonstrated a good correlation, as depicted in Fig. 2 ▸. The dependence can be described well by a linear relationship between the two parameters with Pearson correlation coefficients *r* = 0.80 for *DAMMIF* models and *r* = 0.86 for *GASBOR* models. Separate linear regression models for bead and dummy-residue ensembles based on benchmark data were employed to predict the SAS resolutions from the ensemble variation Δ_ens_. The observed correlation between Δ_ens_ and Δ_CC_ allows one to directly estimate the resolution of *ab initio* models from the variation of the ensembles Δ_ens_ as
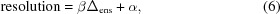
where the coefficient β is the slope presenting the expected change in resolution in response to the change in ensemble variability Δ_ens_, and α is a constant representing the attainable resolution limit at zero variability. Coefficients β of 0.96 ± 0.07 and 1.10 ± 0.09 and constant values α of (7.7 ± 1.3) Å and (5.8 ± 1.2) Å were found for *DAMMIF* and *GASBOR* models, respectively. The 95% confidence intervals for new observations and the fitted functions were computed using the inverse *t* statistic with *n* − 2 degrees of freedom, with *n* being the number of data pairs (Fig. 2 ▸). All statistical analyses were performed with *MATLAB* (Mathworks Inc.).

The ensemble variability is systematically smaller than the actual resolution of the models because of the constraints such as interconnectivity and compactness imposed in the *ab initio* modelling. The constraints restrict the available conformational space, thus increasing the consistency of the models and decreasing the ensemble variation (§S2, Supporting Information). It must be underlined, however, that these constraints are very mild, are always applied in a pre-defined way and do not introduce misfitting to data or inaccuracy in the resulting models. Thus, the established relations between variability and resolution remain constant from one reconstruction to another. Interestingly, both for bead and DR modelling, the linear dependencies are offset by a constant (α), which may be rationalized as the variability that is always present even for idealized cases, *i.e.* the best resolution attainable for SAS-based *ab initio* shape reconstruction (about 7–8 Å for bead modelling and 5–6 Å for DR modelling). The different offset values also correlate well with the fact that the size of the smallest volume element in a structural model limits the maximum obtainable resolution and that bead models have a more coarse-grained representation than dummy-residue models. We should note that the different representations of the hydration layer employed by *DAMMIF* (hydration layer included in bead models) and *GASBOR* (explicit dummy water molecules) have only a minor impact on the relation between variability and resolution.

For the bead modelling, a few data points are observed to fall outside the 95% confidence interval (Fig. 2 ▸ *a*). All of these are structures of oligomeric proteins with internal cavities or holes (§S3, Supporting Information). The bead-modelling procedure always attempts to build the lowest complexity models compatible with the experimental data and therefore tends to smear the finer details. This naturally increases the Δ_CC_ value and explains the elevated Δ_CC_/Δ_ens_ ratio for such structures. Interestingly, the effect is absent for DR-based modelling, which utilizes higher angle data and thus can better represent more complicated shapes (no outliers are observed in Fig. 2 ▸ *b*).

To further validate the proposed method, we performed a jackknife test on 25 synthetic data sets from proteins with known structures taken from the PDB that were not included in the original benchmark set (Fig. 3 ▸ and Supplementary Table S3). Using the variability measure Δ_ens_ of the bead and DR model ensembles, we predicted the effective resolutions with (6) and compared these with the cross-correlated resolution values Δ_CC_. The comparison yielded an excellent correlation, with an *r* of 0.84 and 0.97 for bead and DR models, respectively, indicating high fidelity of the computation of resolution through the variability.

## 4. Implementation and testing of the resolution assessment   

The FSC approach for estimating the resolution of models consists of four steps: (i) pairwise structural alignments of *ab initio* models reconstructed from a given SAS profile, (ii) computation of the scattering amplitudes of the aligned models, (iii) evaluation of the pairwise FSC functions using spherical harmonics and (iv) determination of the model variability and resolution based on the average of the pairwise FSC functions (equation 6 and Supplementary Figs. S1 and S2). Pairs of models are structurally aligned using *SUPCOMB* (Kozin & Svergun, 2001 ▸) or *SUPALM* (Konarev *et al.*, 2006 ▸) and the FSC is computed using spherical harmonics for all possible model pairs within the ensemble. Hence, for an ensemble of *N ab initio* models, *N*(*N* − 1)/2 FSC comparisons are performed. The average FSC function is computed and smoothed using a sliding window of width Δ*s* = 0.1 Å^−1^, which is comparable to the standard width of the shell in reciprocal space (Δ*s* = 0.08 Å^−1^) used in EM FSC computations (Shaikh *et al.*, 2008 ▸). The variability measure Δ_ens_ is defined as 2π/*s*_ens_, where *s*_ens_ is the momentum-transfer value at which the average FSC falls below 0.5. Similarly, the cross-validated resolution Δ_CC_ of an *ab initio* ensemble is obtained from the FSC comparison against the reference high-resolution structure using the same threshold.

### 4.1. Influence of random noise and data range   

We have further checked the effect of noise in the simulated data on the assessment of resolution (Supplementary Table S4). In order to observe the influence of noise on the ensemble variability Δ_ens_ and the cross-correlated resolution Δ_CC_, we generated synthetic SAXS profiles for three proteins (PDB entries 3lzt, 1wla and 1att) and added 5, 10 or 20% of simulated white noise relative to the scattering intensity *I*(*s*) for each data point (Supplementary Table S4). *Ab initio* modelling and resolution estimations were performed in a standard way using these noisy data sets. Both the Δ_ens_ and Δ_CC_ values were found to be stable against random noise added to the simulated data of up to 20% relative to the scattering intensity. The conclusions about the transformation from variability to resolution therefore remain valid even in the presence of random errors in the experimental data.

To test the dependence of the ensemble resolution on the data range, additional *DAMMIF* modelling runs were performed for 12 proteins from the original benchmark set utilizing data in the *s*_max_·*R*_g_ product range from 5.0 to 9.0. The results, presented in Supplementary Table S5, indicate that the effective resolution provided by the bead-model ensembles is not directly related to the data range used. Some minor improvements were observed in Δ_CC_ for small globular proteins with round shapes upon increasing *s*_max_, but in general the variations were within the significance limits and the correlation between Δ_ens_ and Δ_CC_ stayed as given in (6) for all the data ranges used.

Generally, DR models utilizing higher scattering angles showed better correlation with the crystal structures and yielded better Δ_ens_ and Δ_CC_ values compared with the bead models, especially for smaller proteins (Supplementary Tables S1 and S2). However, DR reconstructions using data up to *s*_max_ = 1.0 Å^−1^ (nominal resolution 6 Å) yielded Δ_ens_ and Δ_CC_ values very similar to those obtained using *s*_max_ = 0.5 Å^−1^ (nominal resolution 12 Å). This (somewhat disappointing but important) finding further confirms that most of the information about the particle shape is concentrated at the very low angles. The models built from the DRs are able to fit higher scattering angles and to provide more detailed shapes, but they do not necessarily yield better reconstructions of the high-resolution structure at resolutions beyond 10 Å. This result should by no means be taken as proof that information about the internal structure is not present in the SAS data, but rather as an indication that the approximations utilized in *ab initio* methods do not extract it. Indeed, the DR modelling approach employs identical residues with averaged scattering form factors, representing a chain-compatible assembly. Upon the addition of *a priori* information, *e.g.* sequence, secondary structure and knowledge-based potentials, scattering at higher angles may be meaningfully interpreted in SAXS-assisted folding approaches (Zheng & Doniach, 2005 ▸; Dos Reis *et al.*, 2011 ▸).

Overall, our results further confirm that the resolution of *ab initio* SAS models is not directly related to the range of the fitted data. For the data ranges employed in the shape determination (about 4–7 Shannon channels for bead models and 10–20 channels for DR models), the resolution can be reliably estimated through variability using equation (6).

### 4.2. Symmetric reconstructions   

Macromolecules and complexes consisting of repeating subunits often build symmetric assemblies. If known, the point symmetry can be directly utilized in *ab initio* shape determination as a hard constraint whereby only the asymmetric unit is restored and the complete shape is constructed by appropriate symmetry operations. Symmetric reconstructions available both for bead (Franke & Svergun, 2009 ▸) and DR (Svergun *et al.*, 2001 ▸) models are often employed, *e.g.* for shape analysis of oligomeric proteins. An important question is: how do symmetry constraints influence the variability and resolution of the shape restoration?

To answer this, we have conducted symmetric bead reconstructions using synthetic data from 40 proteins taken from the PDB, including dimers (point symmetry *P*2), trimers (*P*3), tetramers (*P*222) and hexamers (*P*32 and *P*6) (Supplementary Tables S6, S7 and S8). Imposing symmetry reduces the search space, and one might have expected the symmetric reconstructions to be less variable and to potentially provide better resolution compared with the general case. However, the situation is more complicated because the symmetry also makes the real-space search anisotropic. This is especially critical for anisometric particles and it is known that shapes with incorrect anisometry (prolate *versus* oblate) may be obtained when imposing symmetry (Koch *et al.*, 2003 ▸). Given that the desired anisometry can be used as a restraint in the shape reconstructions, the influence of the anisometry condition was also analysed. In general, symmetry constraints tend to increase (and not decrease) the ensemble variability because of the possibility of obtaining reconstructions with varying orientations of symmetry axes and anisometry directions. This effect is more pronounced for single-axis symmetries (*P*2, *P*3 *etc.*; see Supplementary Tables S6 and S7) and is less pronounced for multiple axes such as *P*222 symmetry (Supplementary Table S8; for the latter case, the ensemble variability is indeed comparable with the validated resolution). As expected, imposing incorrect anisometry (*e.g.* prolate reconstruction of an oblate particle) leads to a significant increase in the Δ_CC_/Δ_ens_ ratio. Similar results were obtained with the DR modelling method.

From the overall comparison of the results, it can be concluded that the Δ_CC_/Δ_ens_ ratios for symmetric reconstructions are either less than or equal to those obtained in the asymmetric shape analysis. The variability of the symmetric ensembles can therefore be utilized for the assessment of the resolution using (2) to obtain a conservative estimate of the actual resolution. If needed, this estimate can always be complemented or validated by calculations performed without symmetry restrictions.

### 4.3. Program implementation and applications   

The algorithm to evaluate the resolution of *ab initio* models has been implemented in a Fortran program called *SASRES*, which aligns multiple *ab initio* models provided by *DAMMIF* or *GASBOR* using *SUPCOMB* (Kozin & Svergun, 2001 ▸) (or its faster implementation *SUPALM*; Konarev *et al.*, 2006 ▸) and computes the FSC functions. The alignment and averaging is a standard final step in the shape determination and, given that *SASRES* is integrated in the workflow, its inclusion does not require any additional effort from the user. *SASRES* can also be used as a standalone program on any set of bead or DR models obtained using alternative procedures.

To test the performance of the approach on real data, we applied the resolution-assessment procedure to ten experimental SAXS data sets taken from the SASBDB (Supplementary Table S9 and Fig. 3 ▸). To cross-validate the resolution estimates, entries were selected for which the scattering computed from the available PDB structures matched the experimental SAXS data well according to χ^2^ statistics. Shapes were generated directly from the experimental data using *DAMMIF* and *GASBOR* and were then analysed using *SASRES*. The resolutions estimated by *SASRES* and the cross-validated resolutions Δ_CC_ agree very well (red dots in Fig. 3 ▸ and Supplementary Table S9), demonstrating the robustness of the method as applied to real experimental data.

## 5. Conclusions   

The macromolecular models obtained by structural methods such as MX and EM are always reported in publications and deposited in public archives (Berman *et al.*, 2003 ▸) along with their resolution, which is an extremely important piece of information related to the model quality. The last decade has witnessed tremendous progress in the field of SAS, which has become a mainstream method in structural biology, thanks to new experimental possibilities and to novel data-interpretation approaches to reconstruct three-dimensional models from one-dimensional SAS data. SAS-generated models are now made available to the community through dedicated archives (Hura *et al.*, 2009 ▸; Valentini *et al.*, 2015 ▸). However, until now, no criteria have been available to quantify the resolution of models constructed from SAS data, making it difficult to meaningfully utilize these models to answer biological questions.

The inherent ambiguity of three-dimensional reconstructions from one-dimensional data is one of the major problems of SAS, as multiple (albeit similar at low resolution) models may be generated yielding essentially the same scattering profile. Here, we demonstrate that this ambiguity may paradoxically be useful in establishing the resolution of *ab initio* shape modelling. A resolution measure based on analysis of the average FSC functions within an ensemble of reconstructions compatible with a given data set is introduced. Using numerous simulated and experimental data sets, it is shown that the resolution of *ab initio* models is directly related to the variability of the models in the ensemble. In standard SAS applications, multiple *ab initio* runs are employed to average the shapes and to find the most probable reconstruction. The latter model by definition has the smallest overall shape discrepancy from the other members of the ensemble. Given that the FSC-based measure reflects an average over this ensemble, the resolution assessment by FSC is to be attributed to the most probable reconstruction, which is also the model typically reported in publications. The FSC-based resolution metric sets up a useful threshold for the analysis of *ab initio* SAS models, stating that structural features finer than the resolution cannot be interpreted with confidence. Of course, one should not forget about the possibility of reconstructing an enantiomorphous model of the object. The enantiomorphs are automatically considered in the alignment and averaging procedures (Kozin & Svergun, 2001 ▸; Volkov & Svergun, 2003 ▸; Konarev *et al.*, 2006 ▸) and, correspondingly, in the analysis of the FSC functions.

The program *SASRES* that evaluates the resolution is seamlessly incorporated into the multiple-model analysis but can also run in a standalone mode. The executable of *SASRES* is free for academic users and can be downloaded with the *ATSAS* software suite as of release 2.8 (http://www.embl-hamburg.de/biosaxs/software.html). *SASRES* is also available for online use at http://www.embl-hamburg.de/biosaxs/atsas-online/sasres.php. We expect FSC-based resolution analysis to become a standard step in *ab initio* modelling and propose that the resolution should be reported in publications and depositions of SAS data and models.

## Supplementary Material

Supplementary Tables and Figures.. DOI: 10.1107/S2052252516016018/hi5642sup1.pdf

Excel spreadsheet document (Supplementary Table S1).. DOI: 10.1107/S2052252516016018/hi5642sup2.xlsx

Excel spreadsheet document (Supplementary Table S2).. DOI: 10.1107/S2052252516016018/hi5642sup3.xlsx
