# Supplementary material for: Resolution of ab initio shapes determined from small-angle scattering
Source: IUCrJ. 2016 Oct 27;3(Pt 6):440–7. doi: 10.1107/S2052252516016018 (PMC5094446; doi:10.1107/S2052252516016018)

IUCrJ

Volume 3 (2016)

Supporting information for article:

Resolution of *ab initio* shapes determined from small-angle scattering

Anne T. Tuukkanen, Gerard J. Kleywegt and Dmitri I. Svergun

S1. Fourier shell correlation: validation of the threshold

To define the FSC threshold value for resolution determination, we generated protein structures (for simplicity, C_α–only models) with randomly displaced atomic positions. Starting from a high-resolution X-ray crystallographic structure, the C_α atom positions of a protein structure were randomly displaced by distances drawn from a Gaussian distribution with a mean μ = 0 Å and standard distribution σ such that 2σ equals the target resolution (Supplementary Figure 3). The randomized structures have average resolutions of 2σ compared to the starting structures and, hence, the randomized magnitude is the variance of the random displacement of atoms corresponding to the target resolution (the positions of C_α atoms distribute around the original positions with the average value of σ). The FSC approach to determine resolution was applied on the randomized ensembles and the obtained resolution values at different threshold values were compared with the target resolutions 2σ. The best correlation between the target resolution and FSC resolution was obtained with a threshold value of 0.5.

S2. Influence of compactness

The ensemble variability Δ_{ens} was found to be consistently smaller than the actual resolution of the models Δ_{CC} due to the constraints such as interconnectivity and compactness applied in the *ab initio* modelling. To further verify this hypothesis, DAMMIF runs on three benchmark proteins (PDB codes: 1IGD, 1WLA, 3LZT) were conducted omitting the compactness requirement. This procedure increased the ensemble variability nearly to the level of actual resolution (1IGD: Δ_{ens} = 19 Å / Δ_{cc} = 22 Å; 1WLA: Δ_{ens} = 30 Å / Δ_{cc} = 30 Å; 3LZT: Δ_{ens} = 24 Å / Δ_{cc} = 27 Å). However, the appearance of the non-compact models is rather unphysical and for all practical purposes running the shape determination with constraints ensuring protein-like models is recommended. The variability Δ_{ens} may then serve as an estimate of the upper limit of resolution. The use of the empirical linear relationship specific for either bead or dummy residue models is the best way to assess the effective resolution of an ensemble.

S3. Outliers in the benchmark protein set

A simple linear model was found to fit the correlation between model variability Δ_{ens} and the corresponding cross-validated resolutions for dummy bead and DR models. In the case of bead models, some outliers were observed outside the 95% confidence intervals for the predicted values

(Figure 2a). We studied the structural features of the benchmark proteins in order to understand the reason for this discrepancy. For this purpose, the solvent-accessible surface area A_{SAS} of each benchmark protein was compared with the surface area A_{Rg} of a corresponding sphere having the same radius of gyration R_g. The A_{SAS} / A_{Rg} ratio reflects the complexity of the shape, with higher ratios indicating the presence of cavities on the protein surface and/or internal holes. All the outlier benchmark proteins (PDB codes: 1FA2, 1OAD, 1UUN, 3V03) were found to have A_{SAS} / A_{Rg} ratios (1FA2: 3.2, 1OAD: 3.4, 1UUN: 3.1, 3V03: 2.8) higher than the average over the entire set of structures (Average A_{SAS} / A_{Rg} = 2.4 ± 0.4). This makes these proteins difficult targets for *ab initio* bead modelling with DAMMIF, which includes a condition of compactness of the restored shape and therefore explains the higher Δ_{cc} values. For the DR modelling with GASBOR, not requiring explicit shape compactness, no outliers were observed (Figure 2b).

S4. The effect of ensemble size and the stability of resolution estimates

We have studied the effect of the number of SAS-based models in an ensemble (Number of models N varied between 2 and 20) on the resolution estimate and found that the resolution values were stable starting from N = 5-10. Furthermore, we tested the stability of resolution estimate by generating two independent ensembles of 20 *ab initio* models based on the same SAXS datasets (PDB codes: 1FA2, 1OAD, 1SCA, 1WLA, 2C10, 3V03, 3VGZ). Standard deviation of resolution estimates in this duplicate analysis was found to be ± 5 %.

Table S1 Statistics of DAMMIF model ensembles reconstructed using data range [0 s_{max}] where s_{max} depends on the product $s_{max} \bullet R_g = 7$. Proteins were selected to represent different SCOPe families and molecular weights. (Separate excel file: hi5642sup4.xlsx)

PDB code	Δ_{ens}, Å	Δ_{CC}, Å	SCOPe id	D_{max}, Å	MW, Da
1A19	11.0	15.3	c.9.1.1	60.25	20641.55
1A6Q	20.9	27.0	d.219.1.1	76.79	42707.93
1AFC	7.9	15.6	b.42.1.1	42.82	134831.23
1ATT	13.1	20.6	e.1.1.1	75.73	97411.82
1B73	29.3	35.6	c.78.2.1	85.73	27978.90
1BHT	15.1	25.7	g.10.1.1	67.80	41383.64
1CY9	32.8	38.0	e.10.1.1	131.10	59319.64
1DOV	23.9	25.3	a.24.9.1	132.14	19876.10
1EMS	32.8	47.8	d.13.1.1	105.30	101375.35
1F1E	23.9	26.5	a.22.1.2	63.59	17250.49
1FA2	26.5	52.2	c.1.8.1	132.80	56516.52
1FS3	13.6	21.2	d.5.1.1	52.41	13708.40
1GN9	13.2	21.4	e.26.1.1	73.19	118631.49
1GQQ	37.1	41.8	c.5.1.1	131.59	104115.45
1GXN	16.7	24.2	a.102.5.1	64.13	36712.00
1H3L	11.4	22.0	a.177.1.1	49.67	20314.71
1H4X	8.3	15.8	c.13.2.1	43.10	26654.89
1HB6	16.9	18.6	a.11.1.1	47.77	10043.79
1HGU	14.3	26.1	a.26.1.1	60.00	21902.95
1HOE	9.8	14.2	b.5.1.1	40.41	7967.80
1IGD	7.7	17.1	d.15.7.1	46.10	6657.42
1JK4	7.2	18.2	b.9.1.1	42.81	10641.02
1KPT	14.7	26.1	d.70.1.1	60.88	22134.81
1KYQ	31.0	37.1	c.2.1.11	86.22	98858.91
1L9L	11.4	15.9	a.64.1.1	42.84	9410.84
1MB8	15.3	25.3	a.40.1.1	66.83	28444.78
1NH1	13.2	21.4	e.45.1.1	69.73	37097.52
1O7Z	17.6	19.0	d.9.1.1	52.14	17274.81
1OAD	16.5	45.2	c.1.15.3	104.20	87472.95
1OGM	13.7	24.9	b.133.1.1	78.52	62445.55

PDB code	Δ_{ens}, Å	Δ_{CC}, Å	SCOPe id	D_{max}, Å	MW, Da
1OKB	15.5	18.2	c.18.1.1	55.95	50831.34
1P4P	16.4	17.2	b.76.1.1	54.90	15653.73
1P90	8.8	16.1	c.55.5.2	47.27	15576.76
1P9Q	22.6	25.7	a.5.8.1	86.65	29177.87
1QGV	15.2	16.7	c.47.1.8	48.13	16807.46
1QOU	18.0	31.5	b.17.1.1	76.80	40700.88
1RC2	16.4	31.0	f.19.1.1	79.78	48605.15
1RWT	23.2	30.4	f.43.1.1	72.93	419139.62
1S7J	11.2	17.6	d.21.1.2	62.97	58759.31
1SCA	13.9	20.1	c.41.1.1	58.23	27409.52
1SEK	27.4	34.8	e.1.1.1	108.90	42273.59
1SRQ	31.0	34.1	e.55.1.1	143.78	156263.25
1T07	11.6	22.3	d.279.1.1	50.52	11261.82
1T1F	14.5	20.6	e.1.1.1	81.04	154522.08
1T5T	33.4	38.0	b.82.7.1	140.00	110301.29
1TF9	13.1	19.7	c.56.5.4	55.87	30212.83
1TQ3	10.4	15.9	b.36.1.1	50.10	12738.19
1UBQ	13.2	13.5	d.15.1.1	45.15	8576.91
1UUN	16.5	38.0	f.6.1.2	110.95	38999.35
1V9E	14.9	21.4	b.74.1.1	59.52	58172.36
1VIA	18.2	28.3	c.37.1.2	70.41	41314.74
1W93	27.9	30.4	b.84.2.1	76.50	61523.78
1WAS	21.2	25.3	a.24.2.1	70.09	16366.35
1WC2	14.7	17.8	b.52.1.1	55.90	20003.89
1WLA	11.5	17.1	a.1.1.2	50.90	17696.24
1WPX	21.7	29.3	b.17.1.1	85.81	72623.13
1XAW	17.8	20.1	h.4.17.1	79.29	16601.41
1XEV	14.0	22.9	b.74.1.1	64.71	117418.71
1ZBP	15.8	26.5	e.61.1.1	65.84	30830.22
2AEK	17.2	22.3	no classification	73.86	88342.94
2BB0	12.7	19.7	b.92.1.10	76.62	91481.42
2C10	23.5	32.1	no classification	119.40	336498.53
2DSV	15.8	21.4	c.1.8.5	70.97	42599.09
2EBF	38.0	39.8	a.296.1.1	110.87	85062.90

PDB code	Δ_{ens}, Å	Δ_{CC}, Å	SCOPe id	D_{max}, Å	MW, Da
2END	13.7	21.7	a.18.1.1	57.29	16104.73
2FXI	12.6	16.9	c.44.1.1	47.09	14918.89
2FZP	8.0	15.6	d.345.1.1	47.58	16172.46
2H1T	19.0	34.1	b.178.1.1	83.01	44271.19
2H5Q	16.9	17.4	no classification	60.00	26740.38
2HW4	12.3	21.7	d.322.1.1	52.74	16951.55
2I5H	22.6	24.9	e.71.1.1	69.63	24454.67
2IJR	27.4	31.0	e.66.1.1	76.61	34164.75
2IVN	16.2	22.0	no classification	74.42	36867.77
2J07	18.0	25.3	a.99.1.1	76.11	49324.68
2O3I	22.3	31.5	e.73.1.1	103.18	86565.78
2P12	22.3	36.3	b.175.1.1	80.07	40916.29
2P2T	10.2	17.6	d.39.1.1	54.44	12258.94
2PQY	13.6	20.4	no classification	57.97	27428.19
2Q72	22.0	29.8	f.54.1.1	75.60	60278.01
2QJL	13.7	14.5	no classification	48.79	11150.20
2UWR	9.4	16.7	g.7.1.3	43.78	9204.48
2V8N	19.9	22.0	no classification	80.65	93062.80
2Z45	27.0	32.8	a.280.1.1	72.79	31156.86
3B6X	12.4	14.9	no classification	44.46	29118.93
3BOE	9.4	17.1	c.154.1.1	51.02	22604.05
3CI0	25.3	31.0	d.24.1.7	84.14	62640.71
3EOX	10.2	19.0	no classification	75.72	42751.46
3FR2	13.2	15.6	no classification	45.86	14914.19
3JTC	18.4	22.9	no classification	65.35	56105.76
3LAS	15.3	31.0	no classification	61.23	36883.17
3LZT	12.1	15.9	d.2.1.2	51.90	14880.40
3SRG	15.3	24.2	no classification	58.46	40531.22
3V03	20.1	38.9	no classification	91.92	133424.72
3VGZ	15.8	23.5	no classification	64.30	155411.53
3VN3	16.5	17.4	no classification	62.04	45050.36
4FFE	13.6	16.4	no classification	60.06	54196.11
4RUV	11.5	13.6	no classification	44.53	13124.75
4RV6	16.1	24.6	no classification	72.98	158773.06

PDB code	Δ_{ens}, Å	Δ_{CC}, Å	SCOPe id	D_{max}, Å	MW, Da
4TLQ	14.9	18.6	no classification	48.12	21861.11
4UDP	15.2	22.6	no classification	77.41	115724.83
4X9P	21.2	27.0	no classification	76.15	39141.16
4XTA	13.7	19.4	no classification	66.11	64062.02
4Z0T	10.2	20.4	no classification	60.40	28183.89
5A47	15.2	20.6	no classification	57.36	22377.24
5AA7	13.0	15.8	no classification	48.61	31213.16
5C4N	17.2	28.3	no classification	69.58	26820.28
5CZY	30.4	33.4	no classification	87.23	56208.21

Table S2 Statistics of GASBOR model ensembles reconstructed using datrange [0 s_{max}] where s_{max} = 0.5 Å^{-1}. Proteins were selected to represent different SCOPe families and molecular weights. (Separate excel file: hi5642sup5.xlsx)

PDB code	Δ_{ens}, Å	Δ_{CC}, Å	SCOPe id	D_{max}, Å	MW, Da
1A19	15.9	24.6	c.9.1.1	60.25	20641.55
1A6Q	15.6	27.9	d.219.1.1	76.79	42707.93
1AFC	10.2	14.9	b.42.1.1	42.82	134831.23
1ATT	19.2	20.4	e.1.1.1	75.73	97411.82
1B73	26.1	33.4	c.78.2.1	85.73	27978.90
1BHT	22.0	24.9	g.10.1.1	67.80	41383.64
1CY9	38.0	38.0	e.10.1.1	131.10	59319.64
1DOV	26.1	28.3	a.24.9.1	132.14	19876.10
1EMS	37.1	49.2	d.13.1.1	105.30	101375.35
1F1E	15.1	23.9	a.22.1.2	63.59	17250.49
1FA2	47.8	53.9	c.1.8.1	132.80	56516.52
1FS3	11.1	17.8	d.5.1.1	52.41	13708.40
1GN9	30.4	45.2	e.26.1.1	73.19	118631.49
1GQQ	40.8	46.4	c.5.1.1	131.59	104115.45
1GXN	16.1	26.1	a.102.5.1	64.13	36712.00
1H3L	9.7	18.6	a.177.1.1	49.67	20314.71
1H4X	9.9	15.2	c.13.2.1	43.10	26654.89
1HB6	10.5	18.4	a.11.1.1	47.77	10043.79
1HGU	13.4	27.9	a.26.1.1	60.00	21902.95
1HOE	9.0	14.7	b.5.1.1	40.41	7967.80
1IGD	9.8	19.2	d.15.7.1	46.10	6657.42
1JK4	9.0	14.7	b.9.1.1	42.81	10641.02
1KPT	12.9	24.2	d.70.1.1	60.88	22134.81
1KYQ	26.5	38.0	c.2.1.11	86.22	98858.91
1L9L	10.7	13.6	a.64.1.1	42.84	9410.84
1MB8	15.2	26.5	a.40.1.1	66.83	28444.78
1NH1	15.2	23.2	e.45.1.1	69.73	37097.52
1O7Z	15.9	18.6	d.9.1.1	52.14	17274.81

PDB code	Δ_{ens}, Å	Δ_{CC}, Å	SCOPe id	D_{max}, Å	MW, Da
1OAD	22.0	38.9	c.1.15.3	104.20	87472.95
1OGM	17.2	26.5	b.133.1.1	78.52	62445.55
1OKB	14.3	18.2	c.18.1.1	55.95	50831.34
1P4P	13.6	16.4	b.76.1.1	54.90	15653.73
1P90	10.2	14.2	c.55.5.2	47.27	15576.76
1P9Q	20.4	24.2	a.5.8.1	86.65	29177.87
1QGV	10.9	17.1	c.47.1.8	48.13	16807.46
1QOU	17.1	29.8	b.17.1.1	76.80	40700.88
1RC2	18.6	27.9	f.19.1.1	79.78	48605.15
1RWT	23.5	29.3	f.43.1.1	72.93	419139.62
1S7J	14.8	25.3	d.21.1.2	62.97	58759.31
1SCA	13.5	19.2	c.41.1.1	58.23	27409.52
1SEK	26.1	31.5	e.1.1.1	108.90	42273.59
1SRQ	34.1	33.4	e.55.1.1	143.78	156263.25
1T07	10.4	20.6	d.279.1.1	50.52	11261.82
1T1F	19.0	21.2	e.1.1.1	81.04	154522.08
1T5T	24.2	35.6	b.82.7.1	140.00	110301.29
1TF9	13.5	19.9	c.56.5.4	55.87	30212.83
1TQ3	11.1	17.8	b.36.1.1	50.10	12738.19
1UBQ	11.7	23.2	d.15.1.1	45.15	8576.91
1UUN	37.1	76.0	f.6.1.2	110.95	38999.35
1V9E	15.5	21.2	b.74.1.1	59.52	58172.36
1VIA	16.7	26.5	c.37.1.2	70.41	41314.74
1W93	23.2	27.4	b.84.2.1	76.50	61523.78
1WAS	11.9	23.2	a.24.2.1	70.09	16366.35
1WC2	13.8	17.1	b.52.1.1	55.90	20003.89
1WLA	16.5	17.8	a.1.1.2	50.90	17696.24
1WPX	20.4	27.0	b.17.1.1	85.81	72623.13
1XAW	10.6	19.2	h.4.17.1	79.29	16601.41
1XEV	18.0	22.0	b.74.1.1	64.71	117418.71
1ZBP	15.3	28.3	e.61.1.1	65.84	30830.22
2AEK	16.9	23.5	no classification	73.86	88342.94
2BB0	15.1	21.7	b.92.1.10	76.62	91481.42
2C10	28.8	35.6	no classification	119.40	336498.53

PDB code	Δ_{ens}, Å	Δ_{CC}, Å	SCOPe id	D_{max}, Å	MW, Da
2DSV	19.9	23.2	c.1.8.5	70.97	42599.09
2EBF	36.3	36.3	a.296.1.1	110.87	85062.90
2END	13.8	20.6	a.18.1.1	57.29	16104.73
2FXI	11.8	15.9	c.44.1.1	47.09	14918.89
2FZP	13.1	15.6	d.345.1.1	47.58	16172.46
2H1T	18.4	25.7	b.178.1.1	83.01	44271.19
2H5Q	14.0	15.3	no classification	60.00	26740.38
2HW4	10.3	15.1	d.322.1.1	52.74	16951.55
2I5H	12.5	22.3	e.71.1.1	69.63	24454.67
2IJR	16.4	27.4	e.66.1.1	76.61	34164.75
2IVN	14.8	24.6	no classification	74.42	36867.77
2J07	17.6	25.3	a.99.1.1	76.11	49324.68
2O3I	19.4	29.8	e.73.1.1	103.18	86565.78
2P12	25.7	27.0	b.175.1.1	80.07	40916.29
2P2T	13.7	15.9	d.39.1.1	54.44	12258.94
2PQY	12.9	20.9	no classification	57.97	27428.19
2Q72	21.2	30.4	f.54.1.1	75.60	60278.01
2QJL	10.3	13.4	no classification	48.79	11150.20
2UWR	9.5	18.4	g.7.1.3	43.78	9204.48
2V8N	22.0	23.5	no classification	80.65	93062.80
2Z45	22.6	26.1	a.280.1.1	72.79	31156.86
3B6X	12.3	14.9	no classification	44.46	29118.93
3BOE	14.4	17.6	c.154.1.1	51.02	22604.05
3CI0	19.9	27.4	d.24.1.7	84.14	62640.71
3EOX	17.6	19.2	no classification	75.72	42751.46
3FR2	12.6	13.6	no classification	45.86	14914.19
3JTC	19.0	20.6	no classification	65.35	56105.76
3LAS	15.3	24.6	no classification	61.23	36883.17
3LZT	12.7	15.8	d.2.1.2	51.90	14880.40
3SRG	13.8	24.9	no classification	58.46	40531.22
3V03	29.3	37.1	no classification	91.92	133424.72
3VGZ	16.1	22.9	no classification	64.30	155411.53
3VN3	12.4	15.1	no classification	62.04	45050.36
4FFE	11.9	23.2	no classification	60.06	54196.11

PDB code	Δ_{ens}, Å	Δ_{CC}, Å	SCOPe id	D_{max}, Å	MW, Da
4RUV	10.3	14.3	no classification	44.53	13124.75
4RV6	22.6	27.0	no classification	72.98	158773.06
4TLQ	9.6	14.3	no classification	48.12	21861.11
4UDP	15.9	22.3	no classification	77.41	115724.83
4X9P	15.2	24.6	no classification	76.15	39141.16
4XTA	16.1	18.8	no classification	66.11	64062.02
4Z0T	16.7	20.9	no classification	60.40	28183.89
5A47	13.4	19.9	no classification	57.36	22377.24
5AA7	10.6	16.4	no classification	48.61	31213.16
5C4N	15.8	26.5	no classification	69.58	26820.28
5CZY	28.3	31.5	no classification	87.23	56208.21

Table S3 Resolution estimates for a jack-knife set of DAMMIF and GASBOR *ab initio* reconstructions. The effective resolutions of the ensembles are estimated using the ensemble variability Δ_{ens} and the linear model deduced from the benchmark computations (95% confidence level).

DAMMIF reconstructions

	Δ_{ens},Å	Resolution, Å	Δ_{cc}, Å
5EJW	13.6	21 ± 2	19.9
3KAT	10.2	18 ± 2	13.6
3STK	11.1	18 ± 2	16.1
2AAK	10.4	18 ± 2	15.2
5AG8	14.0	21 ± 2	21.2
1JOM	12.6	20 ± 2	18.0
5DNP	11.7	19 ± 2	17.8
3ATL	11.6	19 ± 2	16.4
1LAF	15.8	23 ± 2	18.0
4Z39	14.5	22 ± 2	19.2
1RLT	11.9	19 ± 2	18.4
5F2T	14.5	22 ± 2	21.7
4UIE	26.1	33 ± 3	27.9
5CZC	14.2	21 ± 2	20.9
1UDT	14.7	22 ± 2	31.5
4Z6G	25.3	32 ± 3	32.1
5DBT	17.1	24 ± 2	21.7
2AET	14.7	22 ± 2	23.2
4UHM	19.0	26 ± 2	24.2
4UYZ	15.8	23 ± 2	22.9
2EJK	14.0	21 ± 2	27.9
5F7C	14.7	22 ± 2	35.6
4L3T	18.8	26 ± 2	30.4
3EJS	21.2	28 ± 2	29.8
5HDT	36.3	43 ± 3	42.9

GASBOR reconstructions

	Δ_{ens}, Å	Resolution, Å	Δ_{cc}, Å
5EJW	14.9	22 ± 3	23.2
3KAT	12.2	19 ± 2	22.6
3STK	12.6	20 ± 2	13.4
2AAK	13.8	21 ± 2	14.9
5AG8	11.8	19 ± 2	20.4
1JOM	16.2	23 ± 3	19.0
5DNP	12.3	19 ± 2	15.9
3ATL	14.0	21 ± 2	16.5
1LAF	13.8	21 ± 2	17.2
4Z39	16.7	24 ± 3	19.4
1RLT	15.3	23 ± 3	27.9
5F2T	16.7	24 ± 3	21.4
4UIE	25.3	34 ± 4	26.5
5CZC	15.9	23 ± 3	22.0
1UDT	18.0	26 ± 3	27.4
4Z6G	27.9	36 ± 4	30.4
5DBT	18.8	27 ± 3	24.2
2AET	19.4	27 ± 3	23.5
4UHM	19.4	27 ± 3	25.7
4UYZ	17.4	25 ± 3	23.9
2EJK	20.6	29 ± 3	27.9
5F7C	19.9	28 ± 3	32.1
4L3T	27.0	36 ± 4	28.8
3EJS	25.3	34 ± 4	30.4
5HDT	41.8	52 ± 5	42.9

Table S4 The effect of signal-to-noise ratio on the variability Δ_{ens} and the cross-validated resolution Δ_{CC}.

Δ_{ens}, Å

PDB code	No noise	5% noise	10% noise	20% noise
1WLA	11.5	11.3	11.4	11.4
1ATT	13.1	14.8	14.7	14.7
3LZT	12.1	10.1	10.3	10.0

Δ_{CC}, Å

PDB code	No noise	5% noise	10% noise	20% noise
1WLA	16.9	16.7	16.6	16.9
1ATT	20.6	21.2	21.2	21.2
3LZT	15.9	16.8	16.2	16.4

Table S5 Effect of the data range used in the modelling process on the Δ_{ens}/Δ_{CC} ratio between the ensemble variation and the cross-validated resolution. The studied data ranges were [0 s_{max}], where $s_{max} \bullet R_g$ equals either 5.0, 7.0 or 9.0 for bead modelling. Two data ranges [0 s_{max}], where s_{max} = 0.5 Å^{-1} or s_{max} = 1.0 Å^{-1}, were employed for dummy residue modelling.

Δ_{ens}/Δ_{CC} ratio

	Data range				
PDB code	$s_{max} \bullet R_g$=5.0	$s_{max} \bullet R_g$=7.0	$s_{max} \bullet R_g$ =9.0	0.5 Å^{-1}	1.0 Å^{-1}
1ATT	1.3	1.6	1.6	1.1	1.0
1FA2	3.2	2.0	1.2	1.1	1.1
1FS3	2.0	1.6	1.6	1.6	1.4
1IGD	1.4	2.2	1.1	2.0	1.5
1OAD	2.2	2.7	1.8	1.8	1.4
1SCA	1.5	1.5	1.5	1.4	1.4
1UBQ	1.4	1.0	1.0	2.0	1.9
1V9E	1.1	1.4	1.2	1.4	1.1
1WC2	1.2	1.2	1.8	1.2	1.1
1WLA	1.3	1.5	1.0	1.1	1.0
2C10	1.4	1.4	1.6	1.2	1.2
3LZT	1.8	1.3	1.3	1.3	1.1
3V03	2.1	1.9	1.2	1.3	1.1
3VGZ	1.4	1.5	1.6	1.4	1.1
4Z0T	1.3	2.0	2.1	1.3	1.1
Average	1.6 ± 0.5	1.6 ± 0.4	1.4 ± 0.3	1.4 ± 0.3	1.2 ± 0.2

Table S6 The effect of applying P2 symmetry with and without anisometry constraints (prolate or oblate) during *ab initio* modelling. The results are shown for DAMMIF models (the upper part of the table) and GASBOR models (the lower part of the table). Simulated data of dimeric proteins were used to compare the variability of reconstruction ensembles generated in either P1 or P2 symmetry. Shape annotations of the proteins are as follows: "_p" = prolate, "_o"=oblate.

DAMMIF & GASBOR reconstructions

PDB	P1		P2		P2 correct anisometry		P2 incorrect anisometry	
codes	Δ_{ens}, Å	Δ_{CC}, Å	Δ_{ens}, Å	Δ_{CC}, Å	Δ_{ens}, Å	Δ_{CC}, Å	Δ_{ens}, Å	Δ_{CC}, Å
1A19_p	11.0	15.3	14.9	15.2	14.3	15.3	13.3	20.1
1CY9_p	32.8	38.0	41.8	39.8	37.1	39.8	41.8	59.7
2H1T_p	19.0	34.1	38.0	42.9	16.5	20.1	23.9	52.2
2P12_p	22.3	36.3	31.0	32.8	21.2	29.8	20.1	39.8
2Z45_p	27.0	32.8	25.7	31.0	28.8	29.3	27.4	37.1
1KPT_p	14.7	26.1	23.9	26.1	13.5	24.2	12.3	28.3
2P2T_p	10.2	17.6	31.0	32.8	10.1	19.0	9.4	19.7
1O7Z_o	17.6	19.0	17.8	18.4	19.0	19.9	17.4	18.8
1BHT_o	15.1	25.7	19.9	24.6	20.6	23.9	22.0	27.0
1KYQ_o	31.0	37.1	37.1	40.8	31.0	38.0	27.0	44.0
**Average Δ_{cc}/Δ_{ens} ratio**		1.4		1.1		1.3		1.7

PDB	P1		P2	
code	Δ_{ens}, Å	Δ_{CC}, Å	Δ_{ens}, Å	Δ_{CC}, Å
1A19_p	15.9	24.6	15.8	15.5
1CY9_p	38.0	38.0	34.8	41.8
2H1T_p	18.4	25.7	17.8	20.1

2P12_p	25.7	27.0	26.5	28.3
2Z45_p	22.6	26.1	23.5	26.5
1KPT_p	12.9	24.2	13.6	24.2
2P2T_p	13.7	15.9	14.5	16.1
1O7Z_o	15.9	18.6	16.9	18.0
1BHT_o	22.0	24.9	22.6	25.3
1KYQ_o	26.5	38.0	29.8	34.8
**Average Δ_{cc}/Δ_{ens} ratio**		1.3		1.2

Table S7 Supporting information The effect of P3 symmetry with and without anisometry constraints on the ensemble variability and resolution. The results are shown for DAMMIF models (the upper part of the table) and GASBOR models (the lower part of the table). Shape annotations of the proteins are as follows: "_p" = prolate. "_o"=oblate.

DAMMIF & GASBOR reconstructions

PDB	P1		P3		P3 correct anisometry		P3 incorrect anisometry	
codes	Δ_{ens}, Å	Δ_{CC}, Å	Δ_{ens},. Å	Δ_{CC}, Å	Δ_{ens},. Å	Δ_{CC}, Å	Δ_{ens}, Å	Δ_{CC}, Å
5a95_o	30.4	38.9	36.3	38.0	22.6	39.8	18.2	47.8
4fci_o	20.1	32.8	17.4	41.8	19.7	31.5	18.6	41.8
4hxq_o	20.1	32.1	19.2	41.8	20.6	31.0	18.6	41.8
4wn0_o	18.4	34.8	20.6	31.0	19.4	28.8	13.2	31.0
**Average Δ_{cc}/Δ_{ens} ratio**		1.6		1.8		1.6		2.4

PDB	P1		P3	
codes	Δ_{ens},. Å	Δ_{CC}, Å	Δ_{ens}, Å	Δ_{CC}, Å
5a95_o	32.1	38.0	34.1	30.4
4fci_o	22.6	33.4	20.1	28.3
4hxq_o	21.4	32.8	21.7	31.0
4wn0_o	24.9	32.8	18.6	36.3
**Average Δ_{cc}/Δ_{ens} ratio**		1.4		1.4

Table S8 The effect of P222 symmetry with and without anisometry constraints on the ensemble variability and resolution. The results are shown for DAMMIF models (the upper part of the table) and GASBOR models (the lower part of the table). Shape annotation of the proteins is as follows: "_p" = prolate. "_o"=oblate. "_e" = equant.

DAMMIF & GASBOR reconstructions

PDB	P1		P222	
codes	Δ_{ens}, Å	Δ_{CC}, Å	Δ_{ens}, Å	Δ_{CC}, Å
1OAD_e	21.7	49.2	21.4	46.4
1ZWK_e	18.8	23.2	14.8	22.6
5DEN_p	36.3	53.9	36.3	53.9
5I01_p	18.6	21.2	18.6	20.6
**Average Δ_{cc}/Δ_{ens} ratio**		1.5		1.6

PDB	P1		P222	
codes	Δ_{ens}, Å	Δ_{CC}, Å	$\Delta_{ens,}$ Å	Δ_{CC}, Å
1OAD_e	23.5	38.9	24.6	42.9
1ZWK_e	17.6	26.1	16.7	25.0
5DEN_p	44.0	50.7	45.2	47.8
5I01_p	18.8	25.3	18.2	18.0
**Average Δ_{cc}/Δ_{ens} ratio**		1.40		1.30

Table S9 Resolution assessment (using linear models with 95% confidence level) of DAMMIF and GASBOR *ab initio* model ensembles based on experimental SAXS data.

DAMMIF reconstructions

Protein	SASBDB id	s_{max}, Å^{-1}	Δ_{ens}, Å	Resolution, Å	Δ_{cc}, Å
Myoglobin	SASDAH2	0.42	10.1	17 ± 2	15.8 ± 0.5
CyaC	SASDAX6	0.47	13.7	21 ± 2	16.1 ± 0.5
Ribonuclease A	SASDAR2	0.44	13.3	20 ± 2	18.8 ± 0.6
Lysozyme	SASDA96	0.3	12.0	19 ± 2	15.5 ± 0.5
Endolyse Complex	SASDAD7	0.18	31.0	37 ±3	34.8 ±1.0
BSA	SASDBT4	0.25	27.0	34 ± 3	38.9 ± 1.2
TGA2	SASDA38	0.21	22.9	30 ± 2	34.1 ± 1.0
ThiM	SASDAX8	0.24	18.0	25 ± 2	31.5 ± 1.0
Aldolase	SASDA68	0.19	28.8	35 ± 3	49.2 ± 1.5
Catalase	SASDA92	0.19	29.8	36 ± 3	46.4 ± 1.4

GASBOR reconstructions

Protein	SASBDB id	s_{max}, Å^{-1}	Δ_{ens}, Å	Resolution, Å	Δ_{cc}, Å
Myoglobin	SASDAH2	0.5	12.4	19 ± 2	14.3 ± 0.4
CyaC	SASDAX6	0.5	11.4	18 ± 2	13.3 ± 0.4
Ribonuclease A	SASDAR2	0.5	10.9	18 ± 2	19.0 ± 0.6
Lysozyme	SASDA96	0.5	12.1	19 ± 2	14.9 ± 0.5
Endolyse Complex	SASDAD7	0.5	34.8	44 ± 5	35.6 ± 1.1
BSA	SASDBT4	0.5	27.0	36 ± 4	38.0 ± 1.1
TGA2	SASDA38	0.5	30.4	40 ± 4	34.8 ± 1.0
ThiM	SASDAX8	0.5	24.2	32 ± 3	32.1 ± 1.0
Aldolase	SASDA68	0.5	36.3	46 ± 5	47.8 ± 1.4
Catalase	SASDA92	0.5	27.0	36 ± 4	44.0 ± 1.3

Figure S1 Benchmarking of the FSC approach using synthetic SAXS data. The program CRYSOL was employed with standard parameters to generate noise-free SAXS profiles which were used for *ab initio* modeling (DAMMIF or GASBOR) runs. The average FSC function was computed based on all pairwise FSC computations.

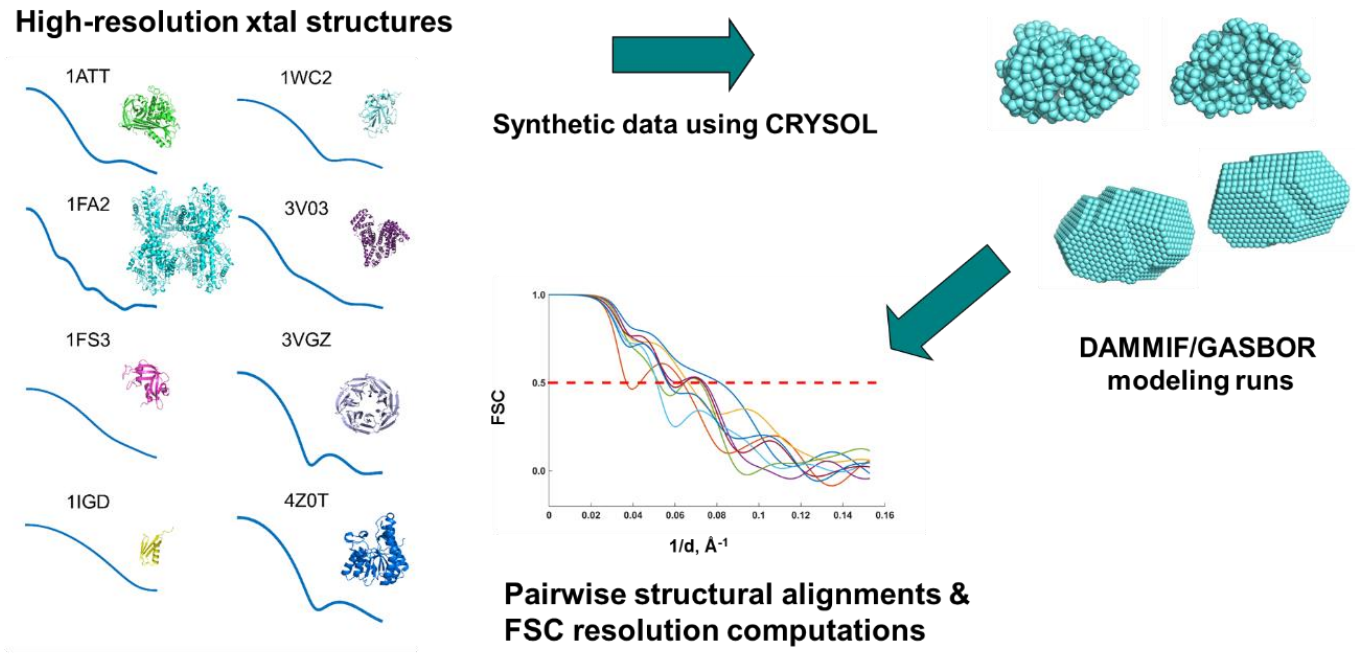

Figure S2 Resolution assessment for SAS-based shape reconstruction of myoglobin. Red, the averaged FSC between the *ab initio* bead models and the high-resolution X-ray crystal structure (PDB code: 1WLA) yielding cross-validated resolution Δ_{cc}; blue, the averaged pairwise FSC between the *ab initio* models providing the variability of the ensemble Δ_{ens}.

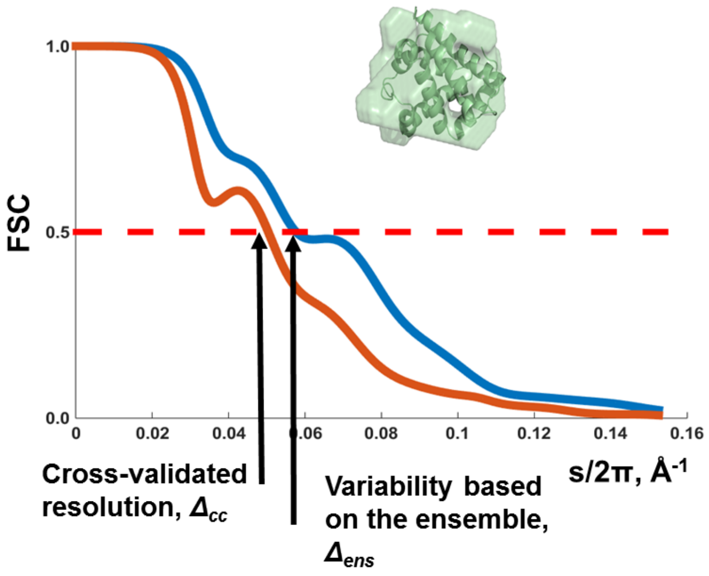

Figure S3 Randomized lysozyme structure (in pink, Cα representation) and the X-ray crystallographic structure (PDB code: 3LZT, in cyan). Uniformly distributed random numbers with mean $\mu = 0$ Å and standard deviation $\sigma = 5.0$ Å were selected to disturb Cα atom coordinates independently in x-, y- and z-directions. Ten randomized structures were generated and the average ensemble resolution was determined using an FSC function threshold value of 0.5. The resolution assessment (11.4 ± 1.2) Å (variation: 9.9 – 13.7 Å) agrees well with the target resolution of $2\sigma = 10.0$ Å

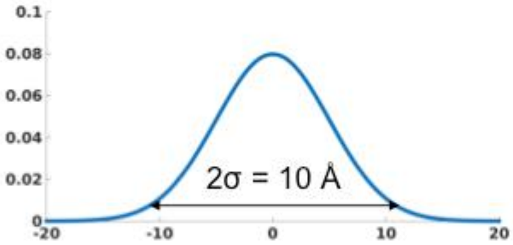

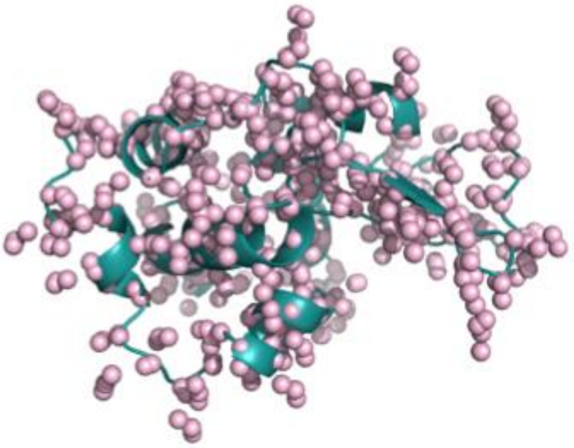

Supplement: Supplementary file 1 [file m-03-00440-sup1.pdf]
